# Feature Extraction for Low-Speed Bearing Fault Diagnosis Based on Spectral Amplitude Modulation and Wavelet Threshold Denoising

**DOI:** 10.3390/s25123782

**Published:** 2025-06-17

**Authors:** Xiaojia Zu, Wenhao Sun, Yuncheng Guo, Yukai Zhao, Haihong Tang, Xue Jiang, Peng Chen

**Affiliations:** 1 School of Marine Engineering Equipment, Zhejiang Ocean University, Zhoushan 316022, China; 2 School of Electronic Information Engineering, Taiyuan University of Science and Technology, Taiyuan 030024, China; 3 Graduate School and Faculty of Bioresources, Mie University, Tus 514-8507, Mie, Japan; 4 School of Naval Architecture and Maritime, Zhejiang Ocean University, Zhoushan 316022, China

**Keywords:** low-speed bearing, fault diagnosis, spectral amplitude modulation, wavelet threshold denoising, feature extraction

## Abstract

To address the issue of difficult extraction of bearing fault features caused by weak fault features and strong environmental noise in low-speed, a low-speed bearing fault diagnosis method based on wavelet threshold denoising and spectral amplitude modulation is proposed. The proposed method effectively overcomes the limitation that the traditional spectral amplitude modulation is greatly affected by noise in low-speed. Firstly, the raw signal is subjected to wavelet threshold denoising to reduce the interference of strong background noise, thereby obtaining the denoised signal. Secondly, the denoised signal is subjected to spectral amplitude modulation to enhance the bearing fault impulses. Finally, the envelope spectrum is normalized to facilitate the visual display of fault feature frequencies. The proposed method is analyzed through simulated and experimental signals in low-speed. The experimental results indicate that the proposed method can reduce noise interference and effectively extract fault features in low-speed.

## 1. Introduction

The wind turbine bearing is one of the crucial components of the wind turbine, and its condition directly affects the normal operation of the entire wind turbine [1]. Bearing faults can lead to serious economic losses and safety accidents [2], especially when the bearing is operating in low-speed (20–600 rpm). Therefore, it is of crucial importance to detect wind turbine bearing faults accurately and promptly to ensure the stable operation of the wind turbine in low-speed [3,4].

Signal processing technology [5] mainly extracts effective fault feature information by analyzing the periodic impact components caused by faults in vibration signals [6,7]. Among the bearing fault diagnosis methods [8,9] based on signal processing, the most renowned one is envelope analysis [10]. Envelope analysis [11] applies bandpass filtering to the raw vibration signal using a band-pass filter [12,13]. Then, the fault signal is effectively separated through envelope demodulation [14]. However, the main problem of envelope analysis is that it is difficult to select the effective frequency band for demodulation [15,16]. To solve this issue, Wang proposed a fault diagnosis method named power spectrum screening combination-gram (Psscgram) and demonstrated its optimal demodulation frequency band extraction capability [17]. Another novel optimal demodulation frequency band selection method for bearing fault diagnosis proposed by Wang is the traversal index enhanced-gram (TIEgram), which can accurately identify the most useful fault information [18]. However, there are two issues in those methods [19,20]: (i) the parameters of the above-mentioned methods need to be set manually, which leads to a lack of self-adaptability [21]; (ii) the high computational complexity may lead to the inability to provide the fault results in a timely manner [22,23].

To address the aforementioned issues, Moshrefzadeh et al. introduced a novel nonlinear filtering method, spectral amplitude modulation (SAM), which automatically extracts fault features without the necessity of pre-defined parameters [24]. With simple calculation procedures and high efficiency, it has become widely applicable. Jiang et al. proposed a tacholess order tracking method based on SAM. This method can effectively extract the instantaneous rotation frequency and the fault information of non-stationary bearings [25]. Xiao proposed an energy-constrained swarm decomposition and adaptive spectral amplitude modulation (ECSWD-ASAM) method, which demonstrates superior performance in compound fault extraction [26]. Ying proposed the Stockwell transform spectral amplitude modulation (STSAM) method, which overcomes the inherent defects of the Fourier transform (FT) in SAM and proves the effectiveness of fault feature extraction through experiments [27]. However, the strong background noise is an obstacle to extracting effective feature frequency, especially applying them into low-speed fault diagnosis [28].

(i) **The difficulties in extracting fault features under strong background noise.** It will amplify the noise interference while highlighting the bearing faults since those methods apply weights to the entire amplitude spectrum, thereby masking the fault information [29,30].
(ii) **The difficulties in extracting weak fault features in low-speed.** The fault features of the bearing are weak and may be masked by other noise signals, resulting in the failure to extract the weak fault features in low-speed.

To address the above issues, a fault diagnosis method for low-speed bearings based on wavelet threshold denoising and SAM is proposed (WTD-SAM). Firstly, this method utilized wavelet threshold denoising to process the raw signal after selecting the denoising parameters to reduce the interference of strong background noise, thereby obtaining the denoised signal. In this paper, the selected wavelet basis functions are db4 and sym2. The selected number of decomposition levels is three to eight layers. The selected thresholds include the rigrsure threshold method, the heursure threshold method, the sqtwolog threshold method, and the minimaxi threshold method. The selected threshold functions are the hard threshold function and the soft threshold function. Secondly, the denoised signal is subjected to SAM to enhance the bearing fault impulses. Finally, the envelope spectrum is normalized to facilitate the visual display of fault feature frequencies. The experimental results show that the proposed method can effectively reduce the noise interference and extract the fault features in low-speed. The main contributions of this article are as follows:

(i) The proposed method solved the limitation that traditional SAM is prone to strong background noise interference. It reduced the noise interference in the raw signal and obtained the denoised signal by performing wavelet threshold denoising on the raw signal, thereby reducing the influence of noise on the low-speed bearing.
(ii) The proposed method performed SAM on the denoised signal to enhance the fault impulses of low-speed bearings, thereby effectively extracting weak fault features from the complex low-speed industrial environment.

The remaining parts of this study are organized as follows:

Section 2 outlines the principles and processes of wavelet threshold denoising and SAM. Section 3 details the complete process of low-speed bearing fault diagnosis based on wavelet threshold denoising and SAM. Section 4 validates the effectiveness of the proposed method using simulation signals. Section 5 validates the effectiveness of the proposed method through the experimental results of actual vibration signals. Section 6 presents the conclusions of this study.

## 2. Theory

### 2.1. Wavelet Threshold Denoising

Traditional signal denoising approaches include moving average and filter denoising methods. However, these traditional techniques have the following drawbacks: (i) These methods operate in either the time domain or the frequency domain. (ii) A single-scale representation of noisy signals is insufficient for extracting important information. The wavelet threshold denoising method employed in this paper offers the following advantages over traditional methods: (i) The wavelet domain is more effective for denoising than the time domain [31]. (ii) The wavelet threshold denoising method exhibits multi-scale and multi-resolution characteristics [32]. Therefore, it can achieve a better denoising effect by removing noise while preserving useful information [33].

**Figure 1:**
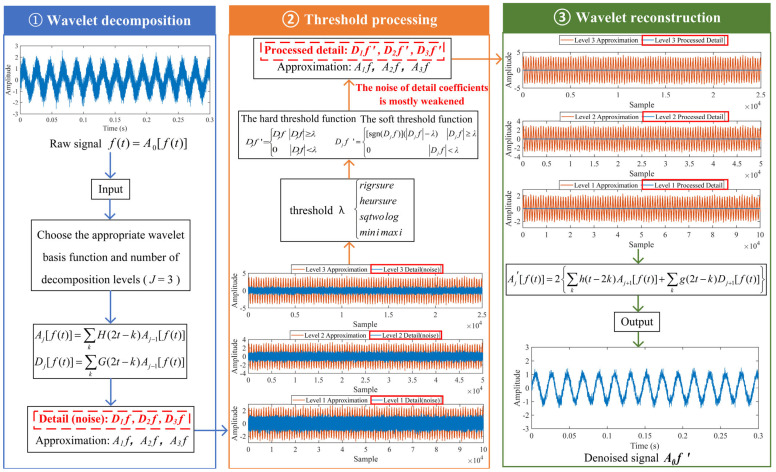
The process of wavelet threshold denoising.

Wavelet threshold denoising consists of three stages: wavelet decomposition, threshold processing, and wavelet reconstruction [34,35]. The process of wavelet threshold denoising is shown in Figure 1. The specific steps of the wavelet threshold denoising process are summarized as follows.

**Firstly, wavelet decomposition.** The appropriate wavelet basis function and number of decomposition levels are selected to perform wavelet decomposition on the raw signal. Wavelet decomposition generates different wavelet coefficients through the Mallat algorithm. The Mallat decomposition algorithm is as follows:

(1) $$\left\{\begin{array}{l} A_{0}[f(t)]=f(t)\\ A_{j}[f(t)]=\sum_{k}H(2t-k)A_{j-1}[f(t)]\\ D_{j}[f(t)]=\sum_{k}G(2t-k)A_{j-1}[f(t)] \end{array}\right.$$

where t is the time series, *t* = 1, 2, …, *N*; *f*(*t*) is the raw signal; *j* is the decomposition level, *j* = 1, 2, …, *J*, *J* is the highest decomposition level; *H* and *G* are wavelet decomposition filters; $A_{j}[\;f\;(t)\;]$ are the approximate part of the wavelet coefficients; $D_{j}[\;f\;(t)\;]$ are the detailed part of the wavelet coefficients; the noise is contained in $D_{j}[\;f\;(t)\;]$; and the useful signal is contained in $A_{j}[\;f\;(t)\;]$.

**Secondly, threshold processing.** Appropriate thresholds and threshold functions are selected to perform threshold processing on the detailed part of wavelet coefficients to obtain the processed detailed part of the wavelet coefficients of each layer. Classical threshold selection methods include the rigrsure threshold method, the heursure threshold method, the sqtwolog threshold method, and the minimaxi threshold method. Commonly used threshold functions are the hard threshold function and the soft threshold function. The hard threshold function is as follows:

(2) $$w_{\lambda 1}=\left\{\begin{array}{l} w\;\left|w\right|\geq \lambda \\ 0\;\left|w\right|<\lambda \end{array}\right.$$

where *w* is the detailed part of wavelet coefficient, λ is the threshold value, and *w*_λ1_ is the wavelet coefficient after hard threshold processing. The soft threshold function is as follows:

(3) $$w_{\lambda 2}=\left\{\begin{array}{l} {}[\mathrm{sgn}(w)](\left|w\right|-\lambda )\;\;\;\left|w\right|\geq \lambda \\ 0\;\;\;\;\;\;\;\left|w\right|<\lambda \end{array}\right.$$

where *w*_λ2_ is the wavelet coefficient after soft threshold processing.

**Thirdly, wavelet reconstruction.** The processed detailed part of the wavelet coefficients of each layer and the approximate part of the wavelet coefficients of the highest decomposition level are used to obtain the denoised signal through the Mallat algorithm. The Mallat reconstruction algorithm is as follows:

(4) $$A_{j}[f(t)]=2\left\{\sum_{k}h(t-2k)A_{j+1}[f(t)]+\sum_{k}g(2t-k)D_{j+1}[f(t)]\right\}$$

where *h* and *g* are wavelet reconstruction filters.

### 2.2. Spectral Amplitude Modulation

**Figure 2:**
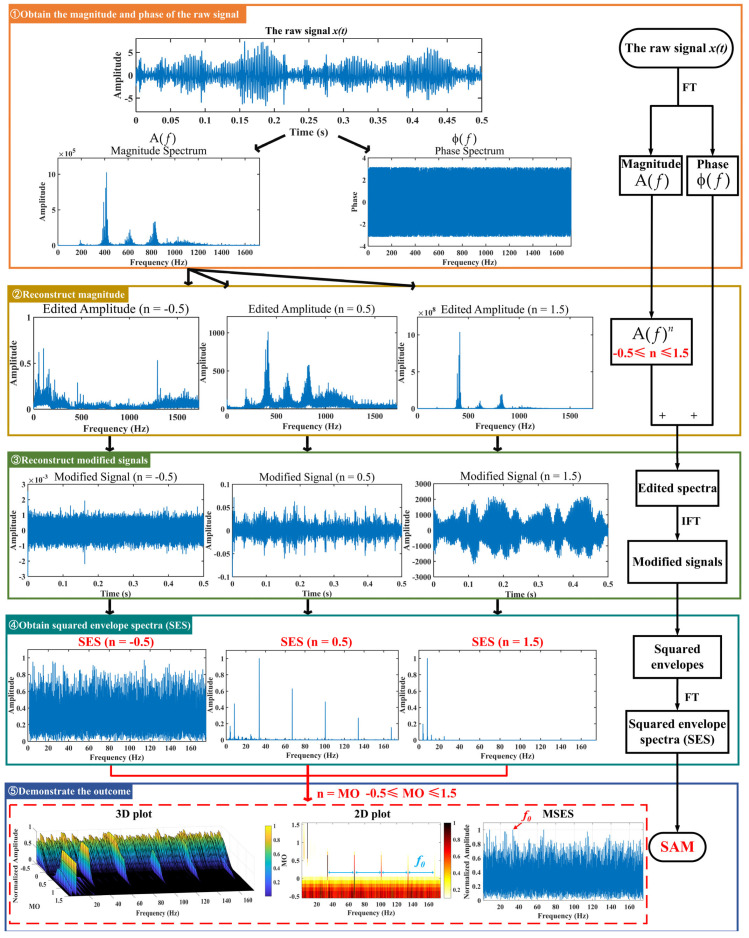
The diagram of SAM.

As a novel nonlinear filtering approach, SAM can automatically decompose signals for different resonance and discrete frequencies based on their amplitudes and extract fault features without the need for pre-defined parameters [36]. It consists of five stages: obtaining the magnitude and phase of the raw signal, reconstructing the magnitude, reconstructing modified signals, obtaining squared envelope spectra (SES), and demonstrating the outcome [37]. The process of SAM is shown in Figure 2. The specific steps of SAM are summarized as follows.

**Firstly, obtain the magnitude and phase of the raw signal.** Let *x*(*t*) be defined as the raw signal. FT is applied to convert the raw signal *x*(*t*) from the time domain to the frequency domain, obtaining the magnitude A( f ) and the phase  ϕ( f ) as follows:

(5) $$X(f)=\mathrm{FT}\{x(t)\}=A(f)e^{j\phi (f)}$$

where *j* is the imaginary unit and FT represents the Fourier transform.

**Secondly, reconstruct the magnitude.** Different weights (MOs) are assigned to the magnitude A( f ) of the signal to adjust the magnitudes of different frequency components. Then, calculate ${A(\;f\;)}^{\mathrm{MO}}$, where MOs range from −0.5 to 1.5 with a step of 0.1. The recommended range of MO is given by ref. [24] as −0.5 to 1.5. The fault pulse signal can be enhanced by appropriately selecting the value of MO. When MO > 1, frequency components with higher magnitudes are further amplified, while frequency components with lower magnitudes are masked, which has a denoising effect. When 0 < MO < 1, as the value of MO decreases from 1 to 0, the dominance of frequency components with larger magnitudes gradually diminishes compared to those with smaller magnitudes. When MO < 0, contrary to the above results, frequency components with lower magnitudes will be amplified.

**Thirdly, reconstruct the modified signals.** SAM assigns different weights to different frequency components, thereby enabling the separation of signals from different sources, which is regarded as a nonlinear filtering process. The actual phase ϕ( f ) of the raw signal and the edited amplitudes ${A(\;f\;)}^{\mathrm{MO}}$ of different weights (MOs) are combined to obtain the edited spectra. The edited spectra are transformed utilizing the inverse Fourier transform (IFT) to obtain the modified signals:

(6) $$x_{m}(t,n)=\mathrm{IFT}\{A{\left(f\right)}^{n}e^{j\phi (f)}\}$$

where $x_{m}(\;t,\;n\;)$ is the modified signal, and the variable *n* is referred to as the Magnitude Order (MO). IFT represents the inverse Fourier transform.

**Fourthly, obtain the SES.** The analytic signal is obtained by performing the Hilbert transform on the modified signal:

(7) $$A\{x_{m}(t,n)\}=x_{m}(t,n)+j*H\{x_{m}(t,n)\}$$

where $A\left\{x_{m}(\;t,\;n\;)\right\}\;$ represents the analytic signal and *H* represents the Hilbert transform. Then, FT is used to calculate the SES as follows:

(8) $$\mathrm{SES}\{x_{m}(t,n)\}=\left|FΤ\{{\left|A\{x_{m}(t,n)\}\right|}^{2}\}\right|$$

**Fifthly, demonstrate the outcome**. The results are normalized within the range of 0 to 1 to compare the SES of different MO. Ultimately, the results of SAM are presented through three-dimensional plots, two-dimensional colormaps, and the Maximum Squared Envelope Spectrum (MSES). In the three-dimensional plot, the x, y, and z axes represent frequency, MOs, and normalized amplitude, respectively. In the two-dimensional colormap, the color is proportional to the normalized amplitude. The x-axis represents frequency, and the y-axis represents MO. The MSES is constructed by selecting the maximum amplitude of each frequency from different MO. In the MSES, the x and y axes represent frequency and normalized amplitude, respectively.

## 3. Proposed Method for Low-Speed Bearing Fault Diagnosis

**Figure 3:**
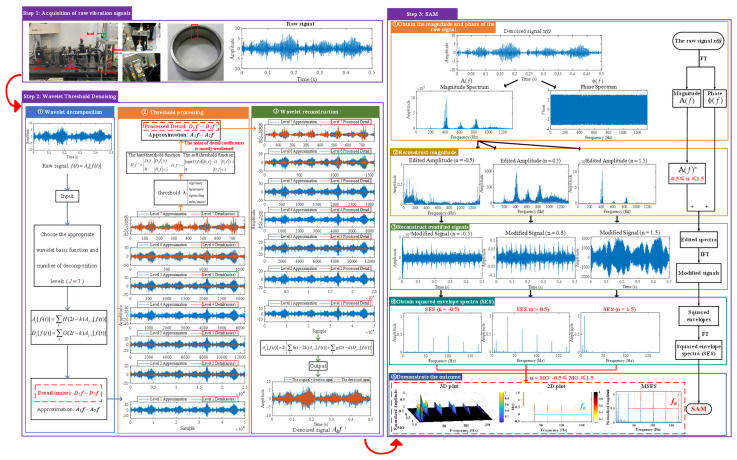
The process of the proposed method.

There are the following drawbacks when the traditional SAM is applied to the raw signal: (i) It is very likely to mask the fault feature frequencies of SAM in a strong background noise environment, thus increasing the difficulty of extracting fault features. (ii) The fault features are weak in low-speed, which makes it difficult for the traditional SAM to extract the weak fault features. As shown in Figure 2, the fault feature frequency, *f*_0_, and its fourth harmonic can be observed only when 0 ≤ MO ≤ 0.7. The fault feature frequency, *f*_0_, cannot be effectively detected in the MSES. To address the above issues, a low-speed bearing fault diagnosis method based on wavelet threshold denoising and SAM is proposed. The proposed method has the following advantages: (i) It reduced the noise interference in the raw signal and obtained the denoised signal by performing wavelet threshold denoising on the raw signal, thereby reducing the influence of noise on the low-speed bearing. (ii) The denoised signal is subjected to SAM to enhance the fault impulses of bearings, thereby effectively extracting weak fault features from the complex low-speed industrial environment. The flowchart of the proposed method is shown in Figure 3, and the pseudo code of the proposed method is shown in Algorithm 1. As shown in Figure 3, when −0.5 ≤ MO ≤ 0.7, not only can the fault feature frequency, *f*_0_, and its fourth harmonic be clearly detected in the 3D and 2D plots, but they can also be distinctly observed in the MSES.
**Algorithm 1: Pseudo code of the proposed method****Input:** raw signal *f*(*t*)**Output:** SAM result chart *Y*
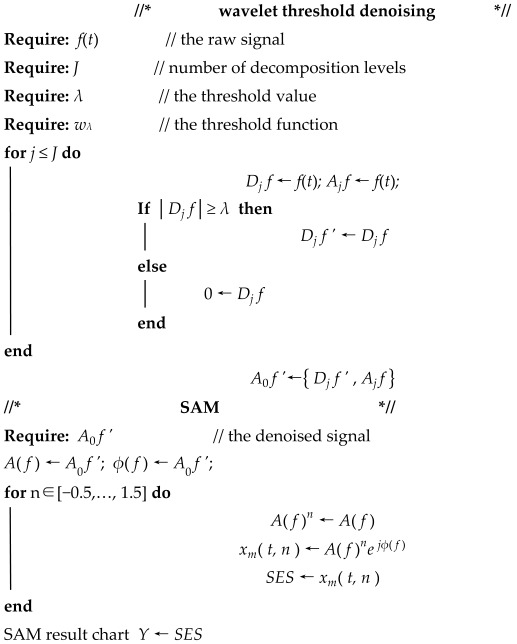


## 4. Simulated Signal Analysis

**Figure 4:**
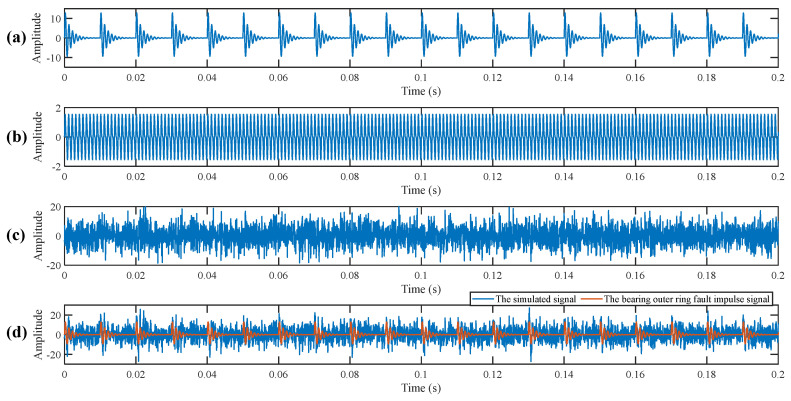
Simulated signal. (**a**) The bearing outer ring fault impulse signal. (**b**) The harmonic interference signal. (**c**) The Gaussian white noise. (**d**) Comparison between the simulated signal and the bearing outer ring fault impulse signal.

**Figure 5:**
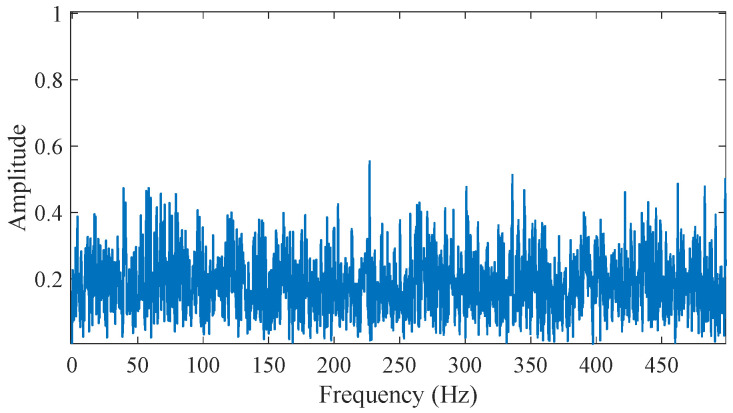
The envelope spectrum of the raw simulated signal.

According to Refs. [38,39], a bearing outer ring fault signal model is constructed to verify the effectiveness of the proposed method. The simulated signal (as shown in Figure 4) consists of three parts: the bearing outer ring fault impulse signal, the harmonic interference signal, and the Gaussian white noise. (i) The bearing outer ring fault impulse signal simulates the periodic impact signal caused by defects in the outer ring of the bearing. (ii) The harmonic interference signal simulates the periodic harmonic interference that is inevitably generated when sensors collect signals. (iii) The Gaussian white noise simulates the background noise under complex industrial conditions. The mathematical expression of the simulated signal is as follows.

(9) $$s_{1}(t)=\sum_{i}A_{i}e^{-2\pi f_{n}g(t-iT)}\sin\left(2\pi f_{n}\sqrt{1-g^{2}}(t-iT)\right)$$

(10) $$s_{2}(t)=P_{1}\sin(2\pi f_{p1}t)+P_{2}\sin(2\pi f_{p2}t)$$

(11) $$x(t)=s_{1}(t)+s_{2}(t)+n(t)$$

where *s*_1_(*t*) represents the bearing outer ring fault impulse signal; *A_i_* represents the amplitude of the ith fault impulse; *f_n_* is the resonant frequency of the fault signal; *g* is the damping coefficient; *T* is the period of the fault signal; the outer ring fault feature frequency *f*_0_ is 100 Hz. *s*_2_(*t*) represents the harmonic interference signal, where *P*_1_ and *P*_2_ are the amplitudes of the harmonic interference signals, and *f_p_*_1_ and *f_p_*_2_ are their frequencies. *n*(*t*) represents Gaussian white noise. The signal-to-noise ratio of the Gaussian white noise is set to −6 dB to simulate the complex working environment of bearings in practical applications. As shown in Figure 4d, the simulated signal contains obvious strong noise interference, and the periodic fault impulse signal becomes less distinct due to the strong noise. The envelope spectrum of the raw simulated signal are shown in Figure 5. There is no obvious fault information in the envelope spectrum.

**Figure 6:**
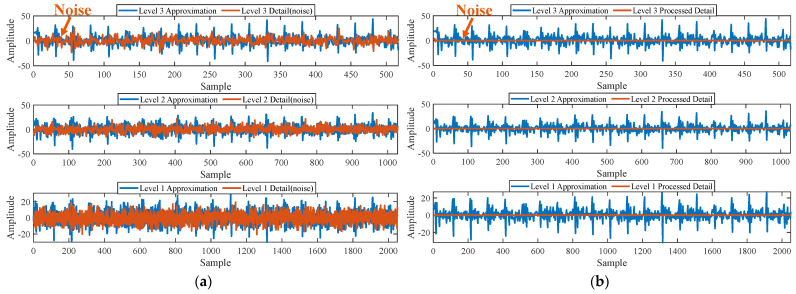
(**a**) The approximate part and the detailed part of the wavelet coefficients at levels 1 to 3. (**b**) The approximate part and the processed detailed part of the wavelet coefficients at levels 1 to 3.

**Figure 7:**
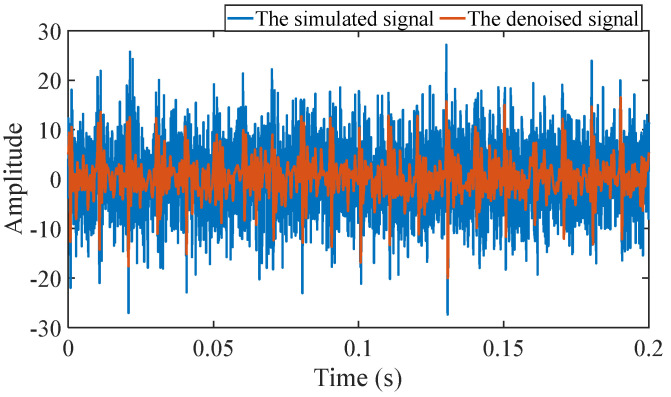
Comparison between the simulated signal and the denoised signal.

**Figure 8:**
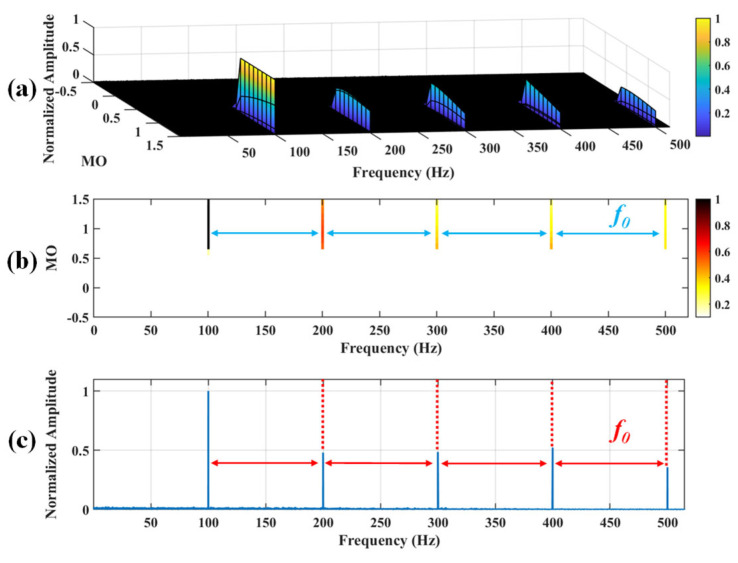
The demonstration of WTD-SAM. (**a**) The 3D plot. (**b**) The 2D plot. (**c**) The MSES.

**Figure 9:**
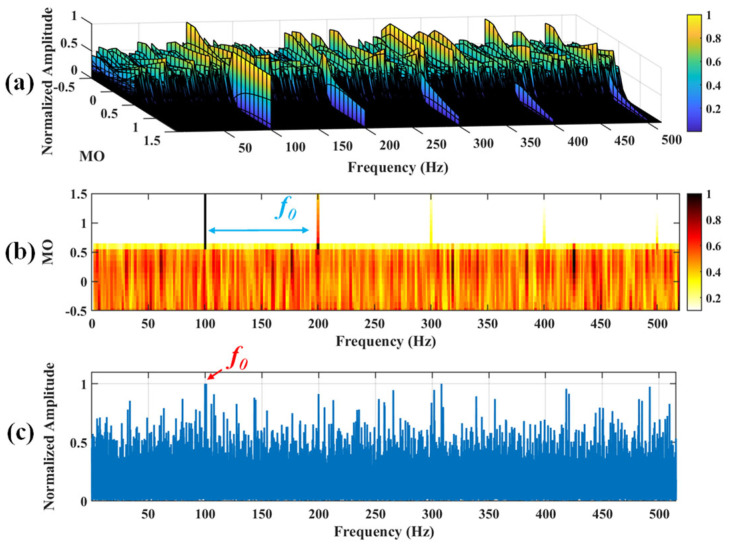
The demonstration of SAM. (**a**) The 3D plot. (**b**) The 2D plot. (**c**) The MSES.

**Figure 10:**
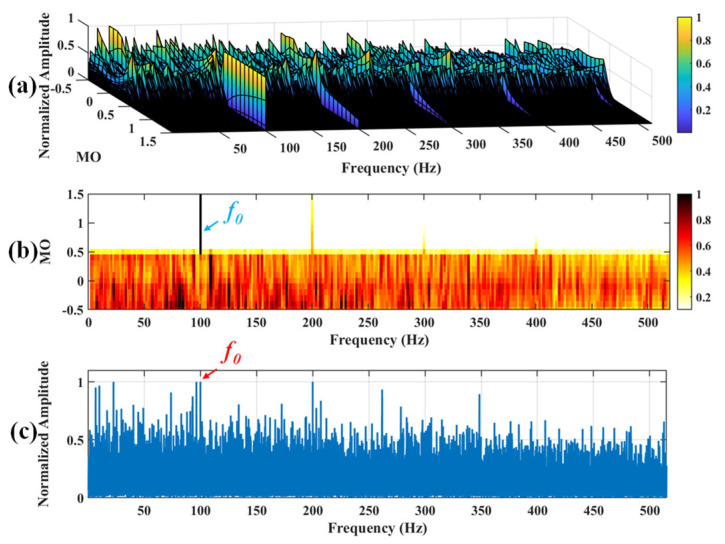
The demonstration of VMD-SAM. (**a**) The 3D plot. (**b**) The 2D plot. (**c**) The MSES.

**Figure 11:**
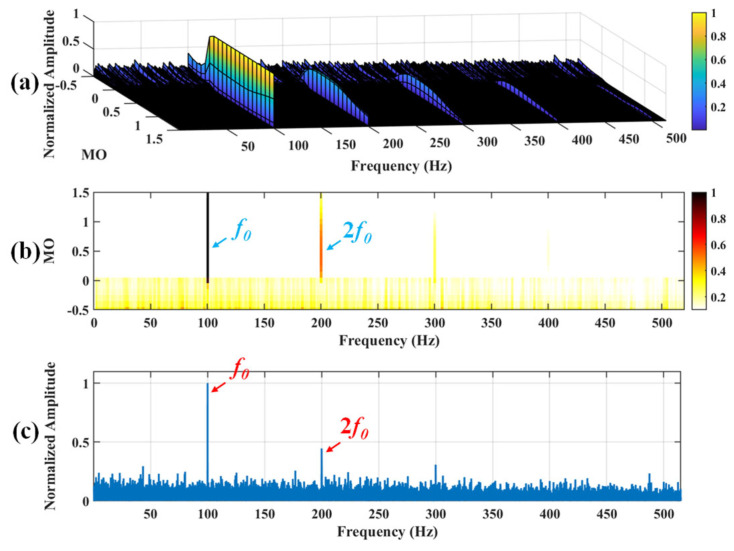
The demonstration of EMD-SAM. (**a**) The 3D plot. (**b**) The 2D plot. (**c**) The MSES.

Firstly, wavelet decomposition is applied to the simulated signal to obtain the approximate and detailed parts of the wavelet coefficients at levels 1 to 3, as shown in Figure 6a. In Figure 6b, the approximate part and the processed detailed part of the wavelet coefficients at levels 1 to 3, which are obtained through threshold processing, are presented. Most of the noise is removed during this process. Subsequently, the denoised signal is obtained through wavelet reconstruction. Figure 7 shows the comparison between the simulated signal and the denoised signal. Distinct periodic impulses can be observed in the denoised signal, and the impact of noise is reduced. Figure 8 presents the results obtained by the proposed method. As shown in Figure 8b, the fault feature frequency, *f*_0_, and its fourth harmonic are clearly visible when 0.6 ≤ MO ≤ 1.5. As shown in Figure 8c, the fault feature frequency, *f*_0_, and its fourth harmonic can be detected in the MSES. Therefore, it can be concluded that this is an outer fault. To validate the effectiveness of the proposed method, the SAM is used to process the simulated signal, and the results are presented in Figure 9. As shown in Figure 9b, only the fault feature frequency, *f*_0_, and its first harmonic can be clearly detected when 0.6 ≤ MO ≤ 1.5. As shown in Figure 9c, the fault feature frequency, *f*_0_, cannot be effectively identified in the MSES. The results obtained by replacing wavelet threshold denoising with variational mode decomposition (VMD) are shown in Figure 10. The results obtained by replacing wavelet threshold denoising with empirical mode decomposition (EMD) are shown in Figure 11. Therefore, based on the above experiments, it can be concluded that the proposed method can effectively identify the fault feature frequency, *f*_0_.

## 5. Experimental Analysis

### 5.1. Case Study A

#### 5.1.1. Experimental Equipment

**Figure 12:**
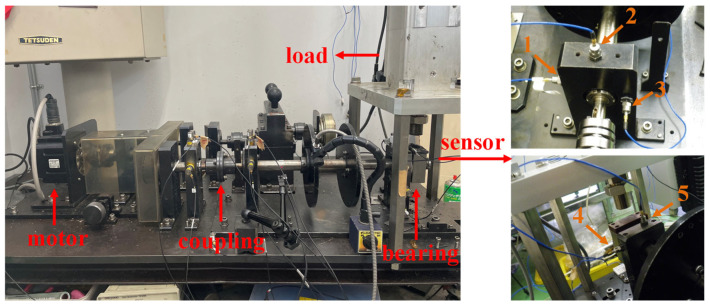
Experimental platforms.

**Figure 13:**
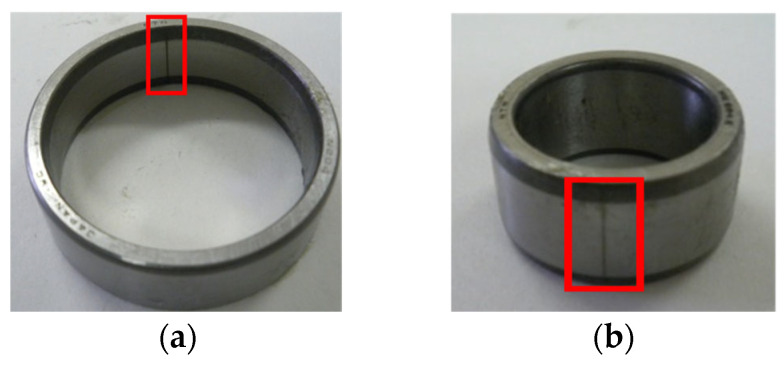
(**a**) Bearing outer fault. (**b**) Bearing inner fault.

**Figure 14:**
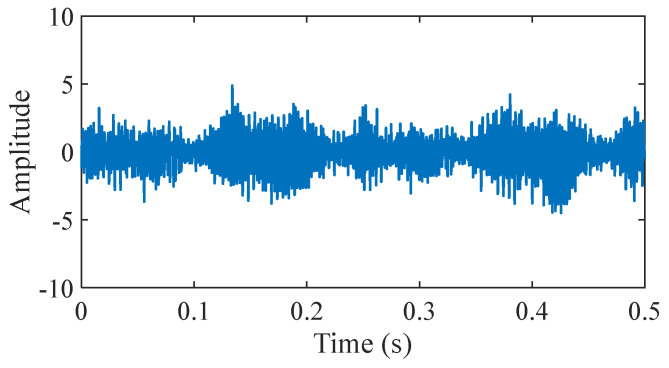
The bearing signal under normal conditions.

As displayed in Figure 12, in this section, the outer fault data and inner fault data collected from the experimental platform are used to verify the application of the proposed method in bearing fault diagnosis at 500 RPM. In this study, the experimental platform produced by Nippon Steel Corporation was employed to illustrate that the signal features of each state are largely congruent with the data features acquired from industrial rotating machinery. To collect more detailed and accurate bearing information, acceleration sensors are placed at different locations to monitor the vibration signals from bearings in different directions or positions. The sensor at position 1 is in the left horizontal direction. The sensor at position 2 is in the left vertical direction. The sensor at position 3 is in the left axial direction. The sensor at position 4 is in the right horizontal direction. The sensor at position 5 is in the right vertical direction. The load is 150 kg. Experimental signals are collected with a sampling frequency of 100 kHz and a sampling time of 20 s. The contact angle is 0 rad. The bearings are shown in Figure 13. The time-domain waveform of the bearing signal under normal conditions is shown in Figure 14. The bearing parameters are displayed in Table 1. The fault feature frequency is shown in Table 2.

#### 5.1.2. Outer Fault

**Figure 15:**
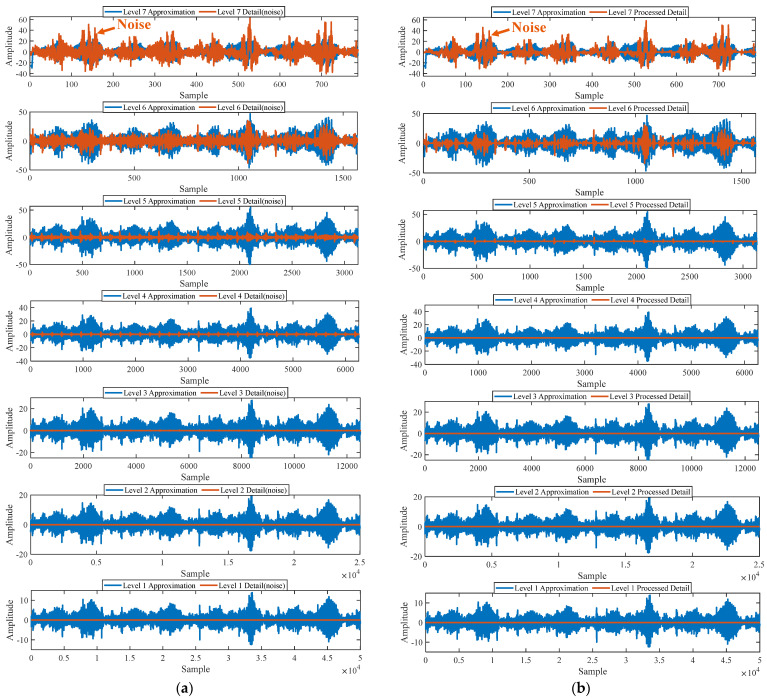
(**a**) The approximate part and the detailed part of the wavelet coefficients at levels 1 to 7. (**b**) The approximate part and the processed detailed part of the wavelet coefficients at levels 1 to 7.

**Figure 16:**
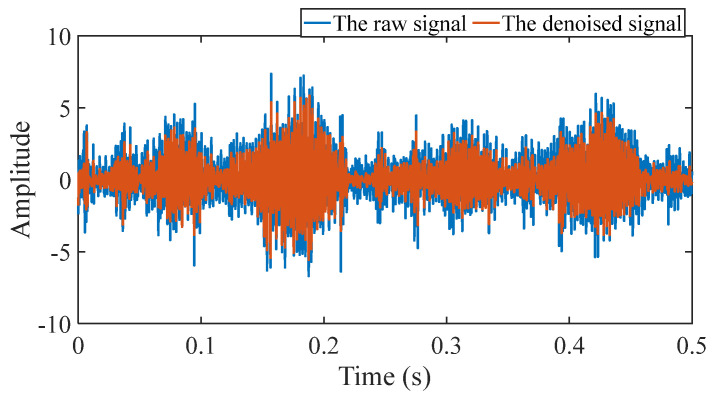
Comparison between the raw signal and the denoised signal.

**Figure 17:**
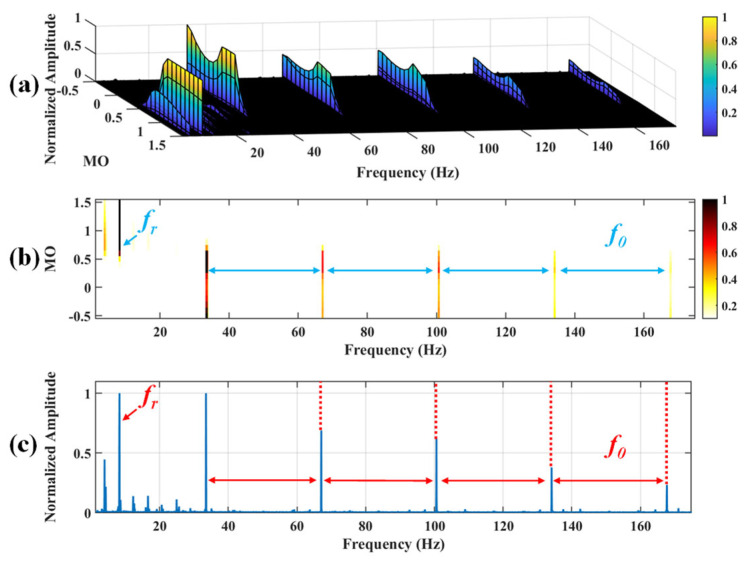
The demonstration of WTD-SAM. (**a**) The 3D plot. (**b**) The 2D plot. (**c**) The MSES.

**Figure 18:**
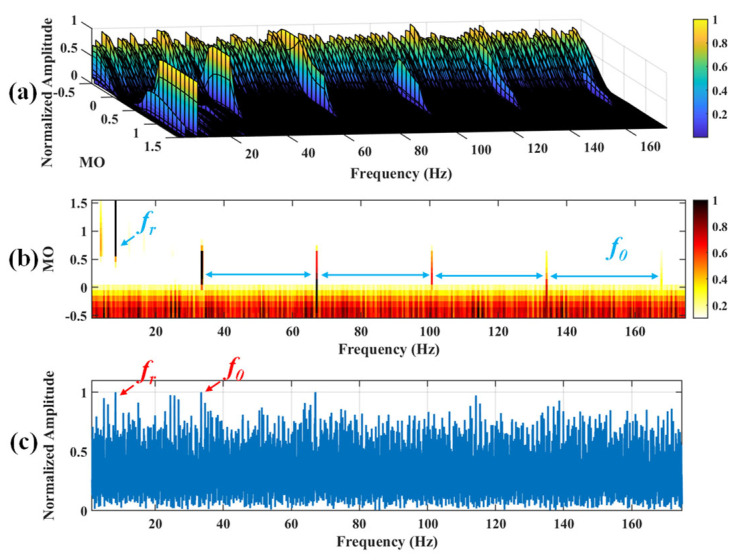
The demonstration of SAM. (**a**) The 3D plot. (**b**) The 2D plot. (**c**) The MSES.

**Figure 19:**
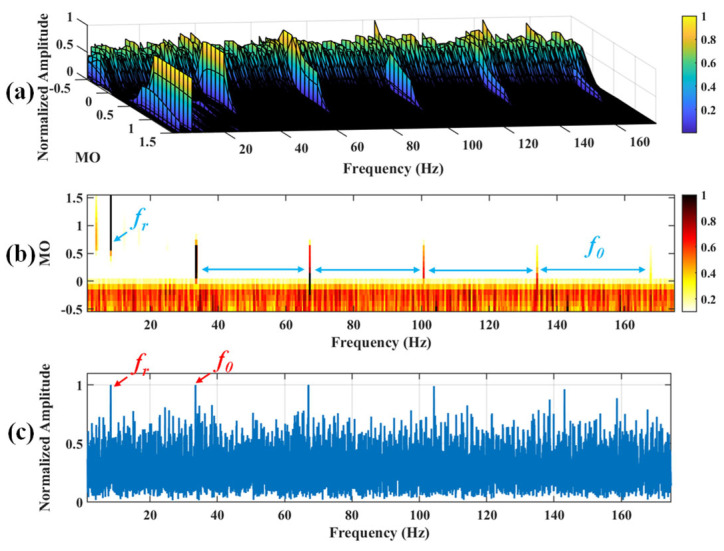
The demonstration of VMD-SAM. (**a**) The 3D plot. (**b**) The 2D plot. (**c**) The MSES.

**Figure 20:**
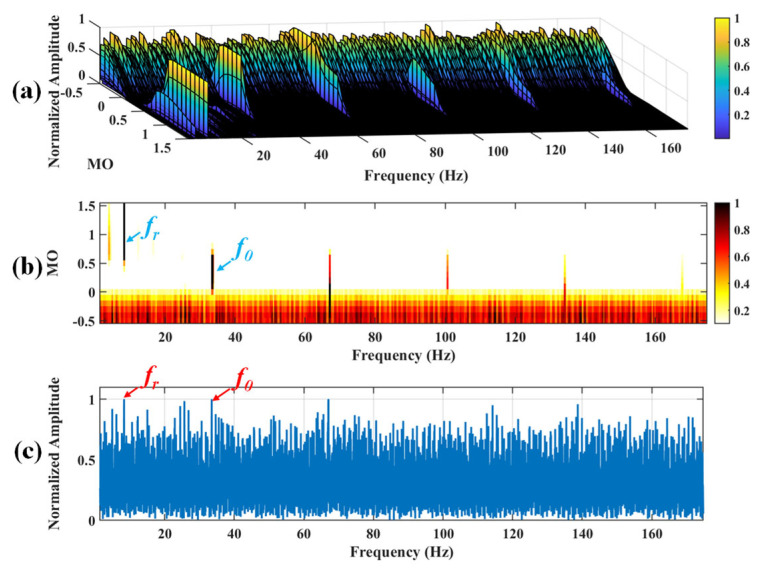
The demonstration of EMD-SAM. (**a**) The 3D plot. (**b**) The 2D plot. (**c**) The MSES.

First of all, wavelet decomposition is applied to the raw signal to obtain the approximate part and the detailed part of the wavelet coefficients at levels 1 to 7, as shown in Figure 15a. Through threshold processing, the approximate part and the processed detailed part of the wavelet coefficients at levels 1 to 7 are obtained, as depicted in Figure 15b. Subsequently, the denoised signal is acquired via wavelet reconstruction. In this experiment, the raw signal is processed using the decomposition level (*J* = 7), the wavelet basis function (db4), and the threshold selection (sqtwolog). The soft thresholding rule is applied at each level. Figure 16 illustrates the comparison between the raw signal and the denoised signal. It can be concluded that the noise interference of the raw signal has been weakened. The results obtained using the proposed method are seen in Figure 17. As seen in Figure 17b, the shaft frequency, *f_r_*, dominates when 0.7 ≤ MO ≤ 1.5. The fault feature frequency, *f*_0_, and its fourth harmonic can be clearly detected when −0.5 ≤ MO ≤ 0.7. In Figure 17c, the shaft frequency, *f_r_*, the fault feature frequency, *f*_0_, and its fourth harmonic can also be clearly observed. Therefore, it can be concluded that there is a fault in the bearing outer ring. The SAM is utilized to process the raw signal, and the corresponding results are displayed in Figure 18. As given in Figure 18b, the shaft frequency, *f_r_*, also dominates when 0.7 ≤ MO ≤ 1.5. The fault feature frequency, *f_0_*, and its fourth harmonic can be observed only when 0 ≤ MO ≤ 0.7. As depicted in Figure 18c, the fault feature frequency, *f*_0_, cannot be effectively detected. The results obtained by replacing wavelet threshold denoising with VMD are shown in Figure 19. The results obtained by replacing wavelet threshold denoising with EMD are shown in Figure 20. Thus, the proposed method can reduce the impact of noise interference and has more advantages in extracting the fault feature frequency, *f*_0_, than SAM.

#### 5.1.3. Inner Fault

**Figure 21:**
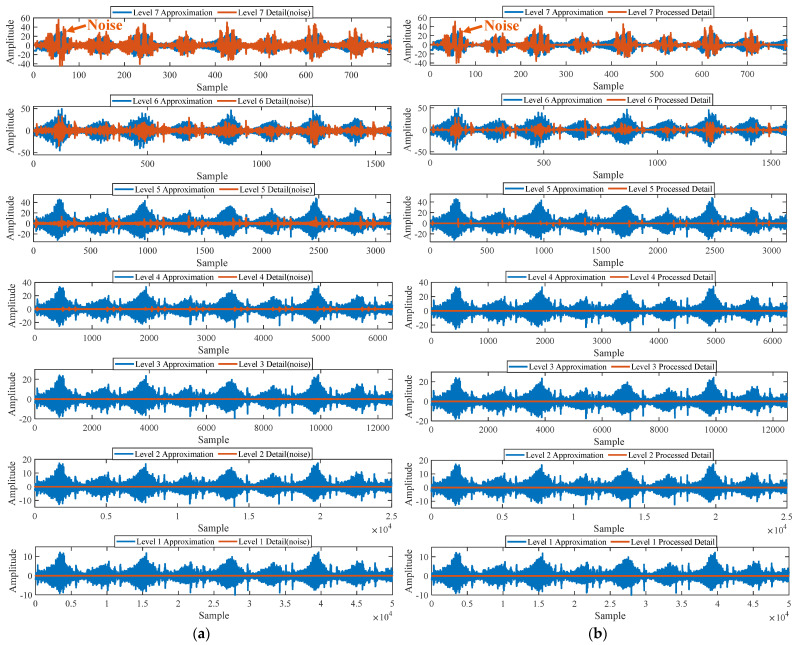
(**a**) The approximate part and the detailed part of the wavelet coefficients at levels 1 to 7. (**b**) The approximate part and the processed detailed part of the wavelet coefficients at levels 1 to 7.

**Figure 22:**
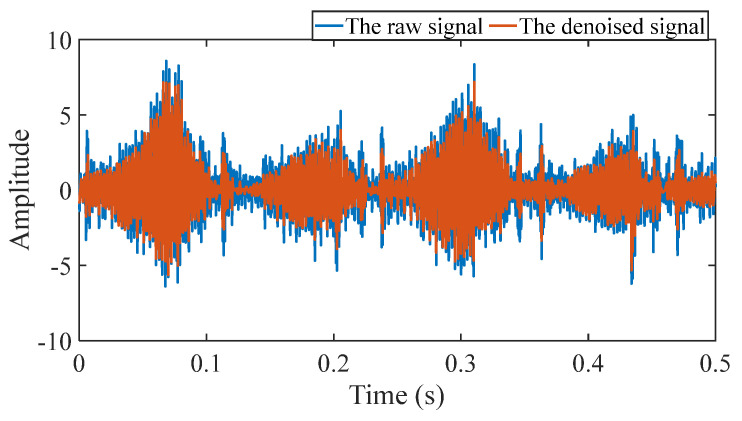
Comparison between the raw signal and the denoised signal.

**Figure 23:**
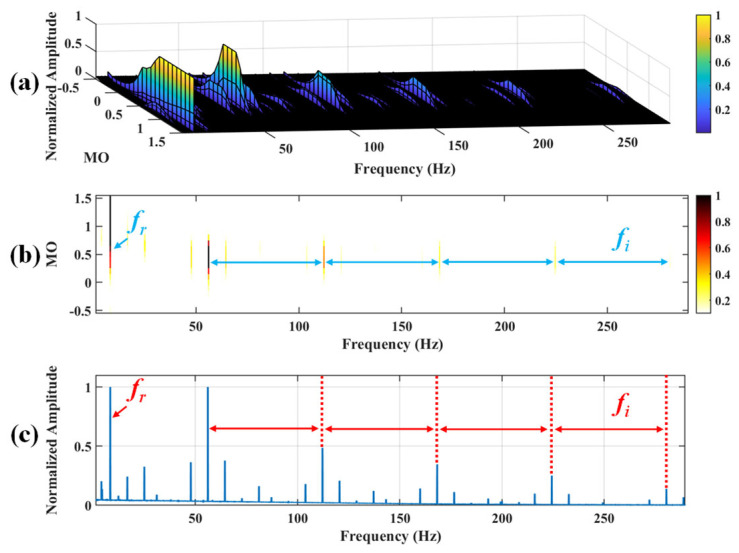
The demonstration of WTD-SAM. (**a**) The 3D plot. (**b**) The 2D plot. (**c**) The MSES.

**Figure 24:**
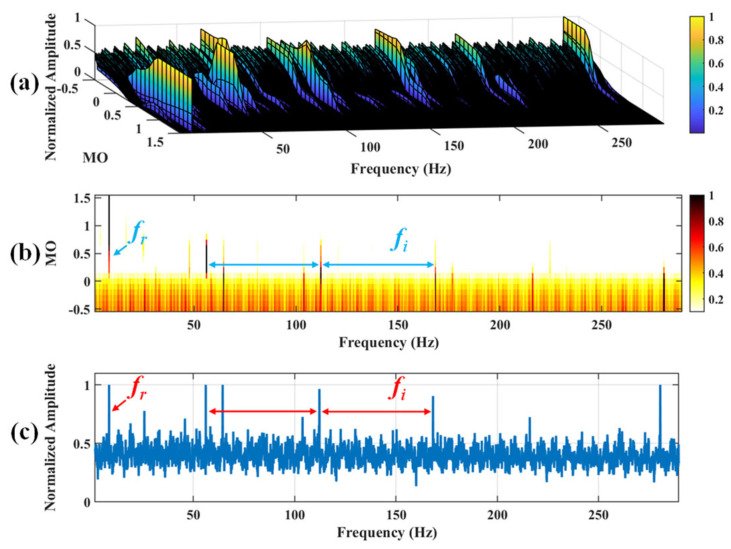
The demonstration of SAM. (**a**) The 3D plot. (**b**) The 2D plot. (**c**) The MSES.

**Figure 25:**
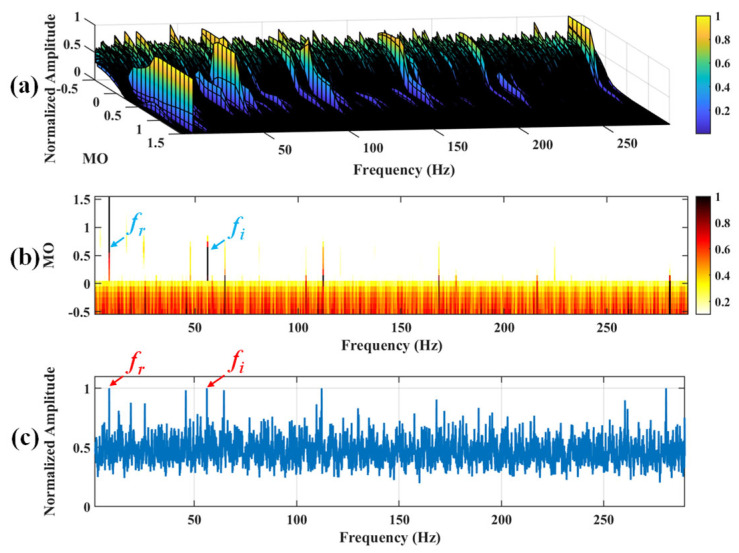
The demonstration of VMD-SAM. (**a**) The 3D plot. (**b**) The 2D plot. (**c**) The MSES.

**Figure 26:**
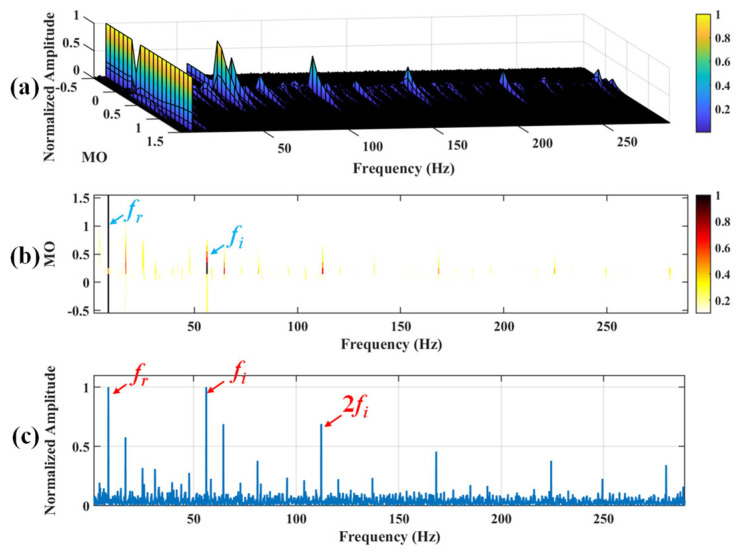
The demonstration of EMD-SAM. (**a**) The 3D plot. (**b**) The 2D plot. (**c**) The MSES.

Firstly, wavelet decomposition is applied to the raw signal to obtain the approximate part and the detailed part of the wavelet coefficients at levels 1 to 7, as seen in Figure 21a. The approximate part and the processed detailed part of the wavelet coefficients at levels 1 to 7 are obtained through threshold processing, as shown in Figure 21b. Subsequently, the denoised signal is obtained through wavelet reconstruction, as shown in Figure 22. In this experiment, the raw signal is processed using the decomposition level (*J* = 7), the wavelet basis function (db4), and the threshold selection (sqtwolog). The soft thresholding rule is applied at each level. Figure 22 shows the comparison between the raw signal and the denoised signal. It can be concluded that the noise interference of the raw signal has been reduced. Subsequently, the results obtained using the proposed method are shown in Figure 23. As seen in Figure 23b, the shaft frequency, *f_r_*, dominates when 0.7 ≤ MO ≤ 1.5. Notably, the fault feature frequency *f_i_* and its fourth harmonic can be detected when 0.1 ≤ MO ≤ 0.8. Additionally, in Figure 23c, the shaft frequency, *f_r_*, the fault feature frequency, *f_i_*, and its fourth harmonic can be clearly observed. For comparison, SAM is utilized to analyze the raw signal, and the corresponding results are shown in Figure 24. As shown in Figure 24b, only the fault feature frequency, *f_i_*, and its second harmonic can be faintly detected when 0.1 ≤ MO ≤ 0.8. As shown in Figure 24c, the fault feature frequency, *f_i_*, cannot be effectively extracted. The results obtained by replacing wavelet threshold denoising with VMD are shown in Figure 25. The results obtained by replacing wavelet threshold denoising with EMD are shown in Figure 26. Thus, the above results indicate that this method can effectively extract the fault feature frequency, *f_i_*.

### 5.2. Case Study B

#### 5.2.1. Experimental Equipment

**Figure 27:**
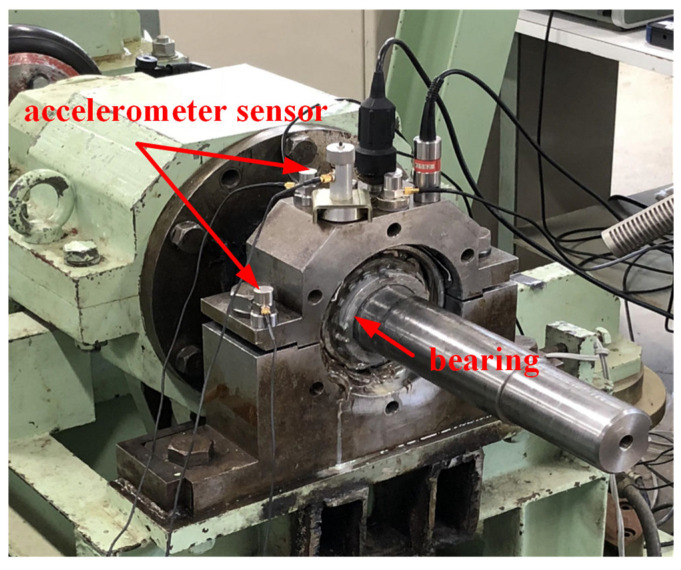
Experimental platforms.

**Figure 28:**
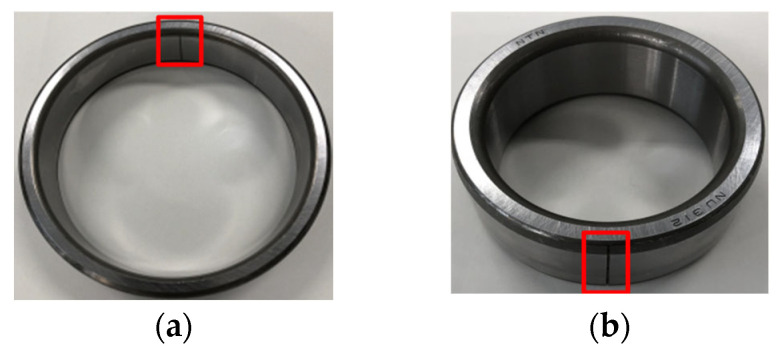
(**a**) Bearing outer fault. (**b**) Bearing inner fault.

**Figure 29:**
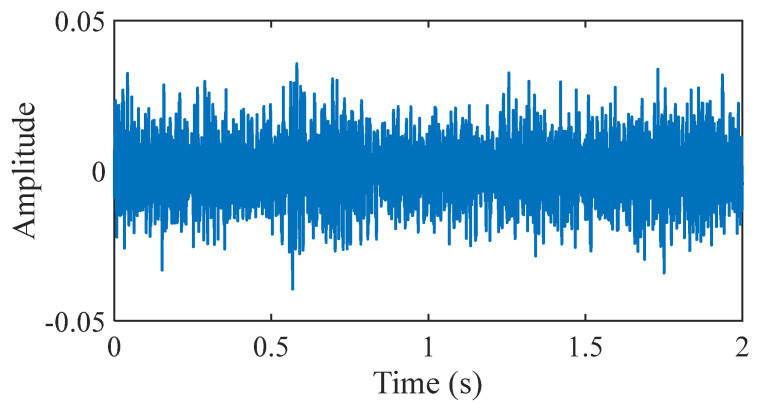
The bearing signal under normal conditions.

This section utilizes the outer fault data and inner fault data collected by the experimental platform shown in Figure 27 to test the application of the proposed method in bearing fault diagnosis at 100 RPM. In this experiment, acceleration sensors are used to collect more detailed and precise bearing information. Experimental data are collected at a sampling frequency of 100 kHz for a duration of 20 s. The bearings are shown in Figure 28. The time-domain waveform of the bearing signal under normal conditions is shown in Figure 29. The bearing parameters are displayed in Table 3. The fault feature frequency is shown in Table 4.

#### 5.2.2. Outer Fault

**Figure 30:**
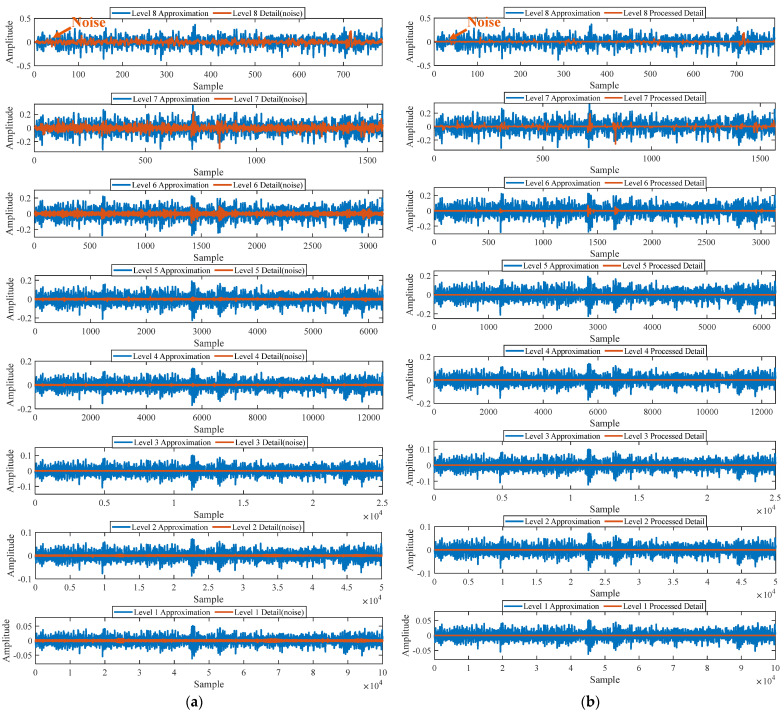
(**a**) The approximate part and the detailed part of the wavelet coefficients at levels 1 to 8. (**b**) The approximate part and the processed detailed part of the wavelet coefficients at levels 1 to 8.

**Figure 31:**
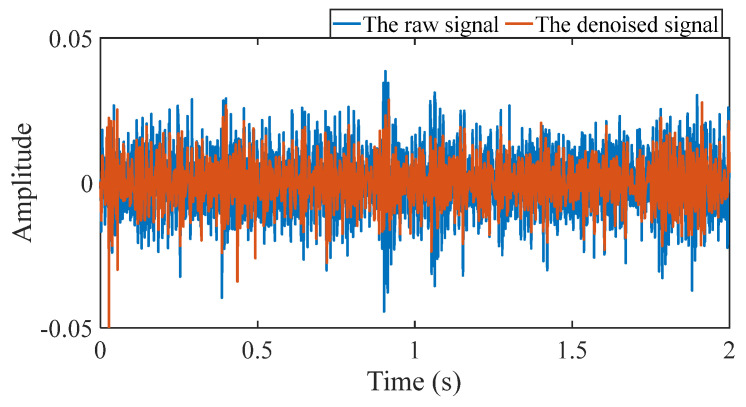
Comparison between the raw signal and the denoised signal.

**Figure 32:**
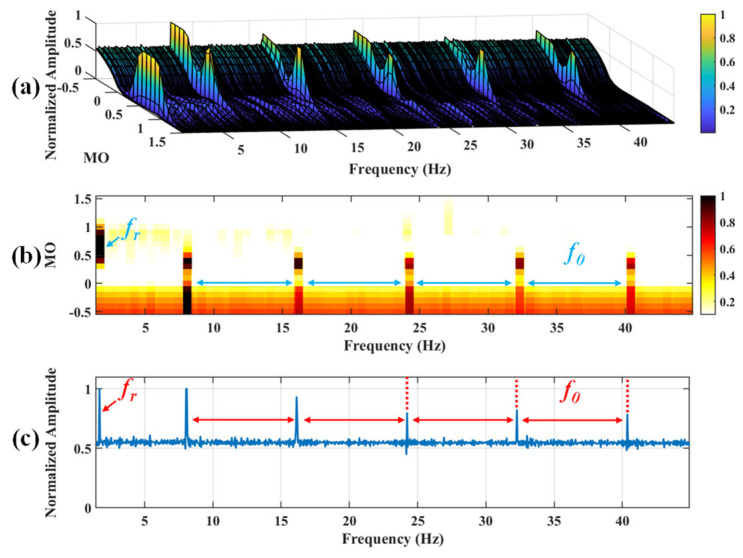
The demonstration of WTD-SAM. (**a**) The 3D plot. (**b**) The 2D plot. (**c**) The MSES.

**Figure 33:**
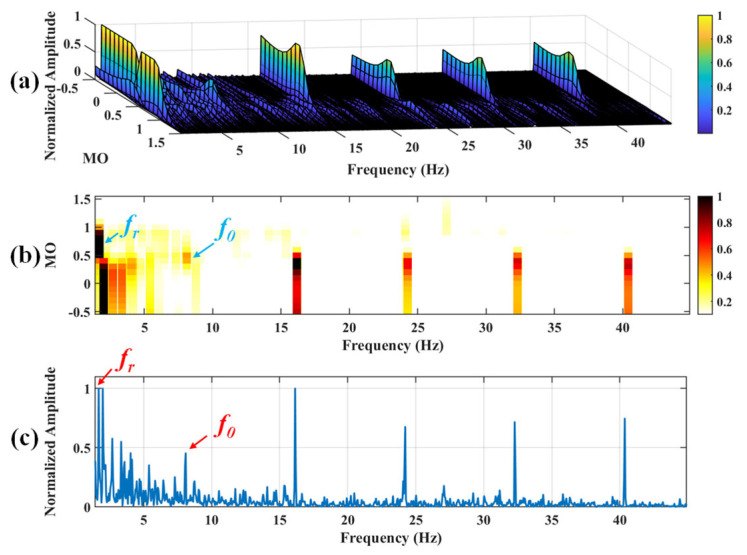
The demonstration of SAM. (**a**) The 3D plot. (**b**) The 2D plot. (**c**) The MSES.

**Figure 34:**
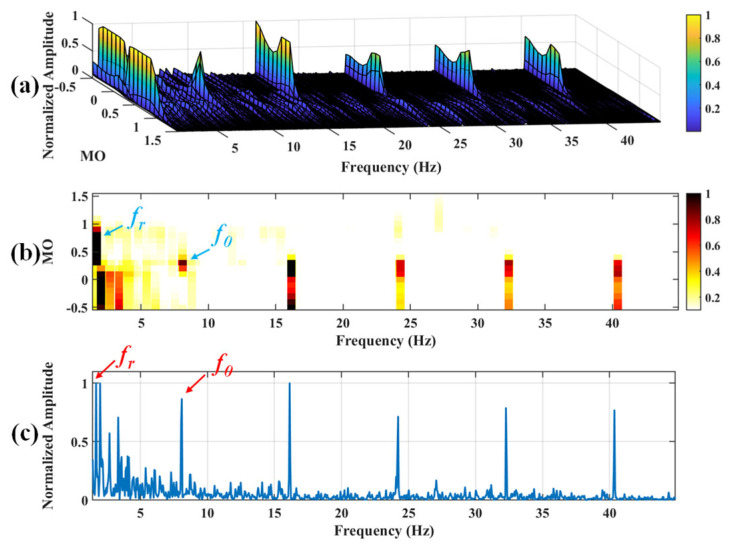
The demonstration of VMD-SAM. (**a**) The 3D plot. (**b**) The 2D plot. (**c**) The MSES.

**Figure 35:**
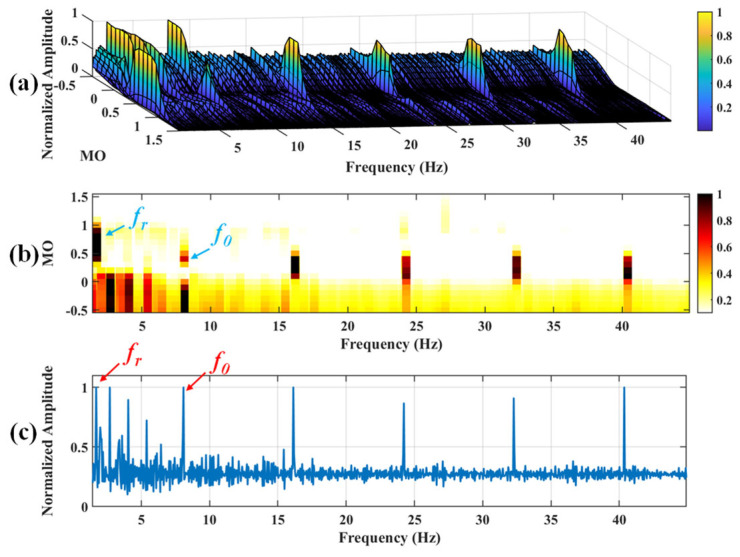
The demonstration of EMD-SAM. (**a**) The 3D plot. (**b**) The 2D plot. (**c**) The MSES.

First, wavelet decomposition is applied to the raw signal to obtain the approximate and detailed parts of the wavelet coefficients at levels 1 to 8, as shown in Figure 30a. The approximate and processed detailed parts of the wavelet coefficients at levels 1 to 8 are obtained through threshold processing, as shown in Figure 30b. Then, the denoised signal is obtained through wavelet reconstruction. In this experiment, the raw signal is processed using the decomposition level (*J* = 8), the wavelet basis function (db4), and the threshold selection (rigrsure). The soft thresholding rule is applied at each level. Figure 31 shows the comparison between the raw signal and the denoised signal. Through this process, the noise interference in the raw signal is reduced. In the following, the proposed method is applied to the signal, as shown in Figure 32. According to Figure 32b, the shaft frequency, *f_r_*, dominates when 0.6 ≤ MO ≤ 1. The fault feature frequency, *f*_0_, and its fourth harmonic can be clearly detected when −0.5 ≤ MO ≤ 0.6. Meanwhile, as illustrated in Figure 32c, the shaft frequency, *f_r_*, the fault feature frequency, *f*_0_, and its fourth harmonic can also be clearly detected, indicating that there is a defect in the bearing outer ring. Figure 33 presents the results of the SAM in processing the raw signal to further validate the applicability of the proposed method. According to Figure 33b, only when 0.5 ≤ MO ≤ 0.6 can the fault feature frequency, *f*_0_, and its fourth harmonic be detected. As shown in Figure 33c, the fault feature frequency, *f*_0_, cannot be effectively extracted. The results obtained by replacing wavelet threshold denoising with VMD are shown in Figure 34. The results obtained by replacing wavelet threshold denoising with EMD are shown in Figure 35. Therefore, based on the above analysis, the proposed method can also effectively extract the fault feature frequency, *f*_0_, when the rotating speed is 100 r/min.

#### 5.2.3. Inner Fault

**Figure 36:**
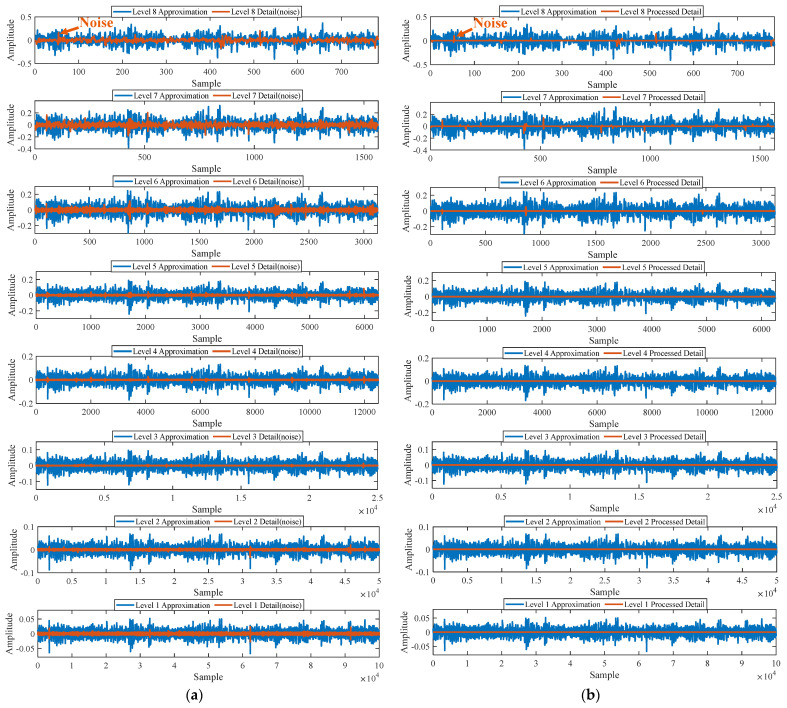
(**a**) The approximate part and the detailed part of the wavelet coefficients at levels 1 to 8. (**b**) The approximate part and the processed detailed part of the wavelet coefficients at levels 1 to 8.

**Figure 37:**
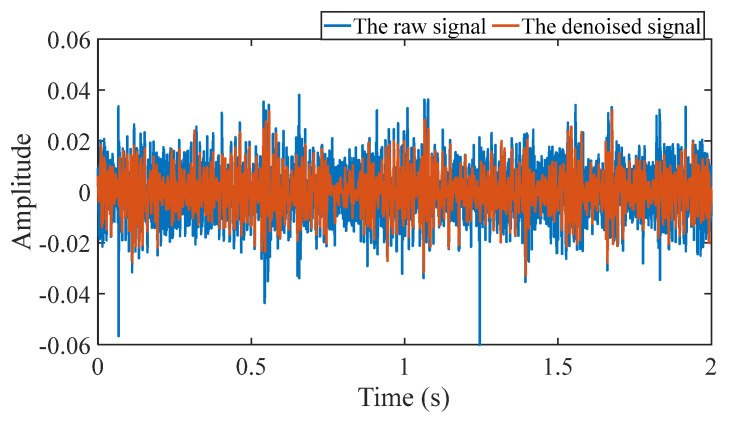
Comparison between the raw signal and the denoised signal.

**Figure 38:**
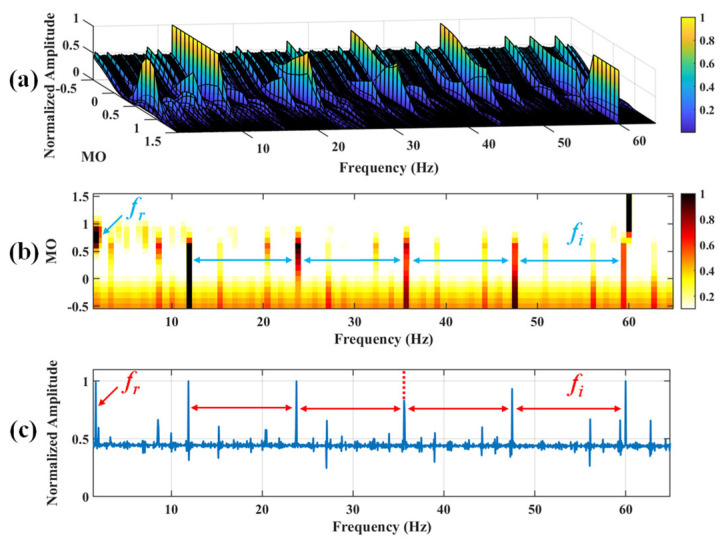
The demonstration of WTD-SAM. (**a**) The 3D plot. (**b**) The 2D plot. (**c**) The MSES.

**Figure 39:**
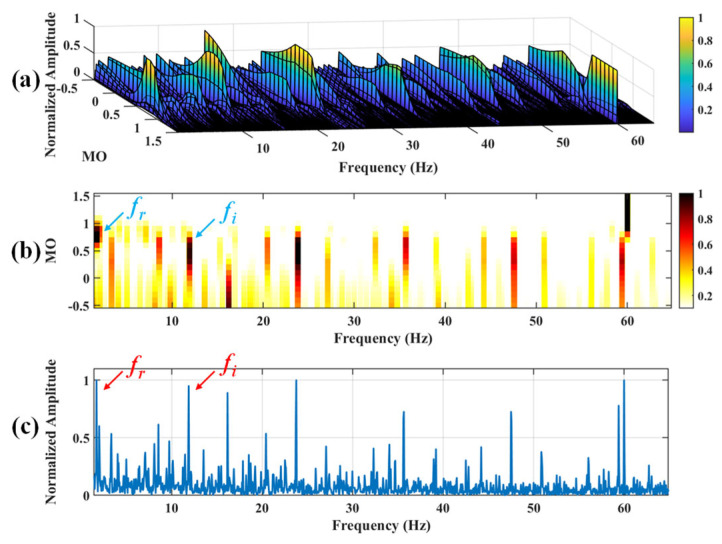
The demonstration of SAM. (**a**) The 3D plot. (**b**) The 2D plot. (**c**) The MSES.

**Figure 40:**
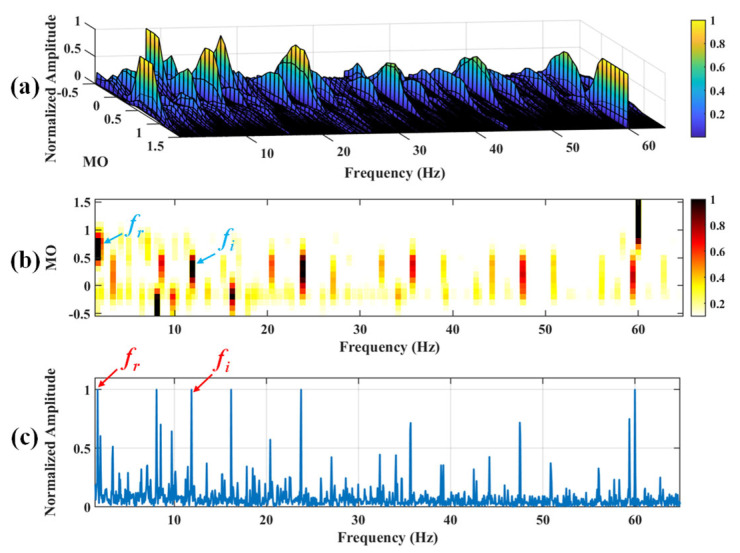
The demonstration of VMD-SAM. (**a**) The 3D plot. (**b**) The 2D plot. (**c**) The MSES.

**Figure 41:**
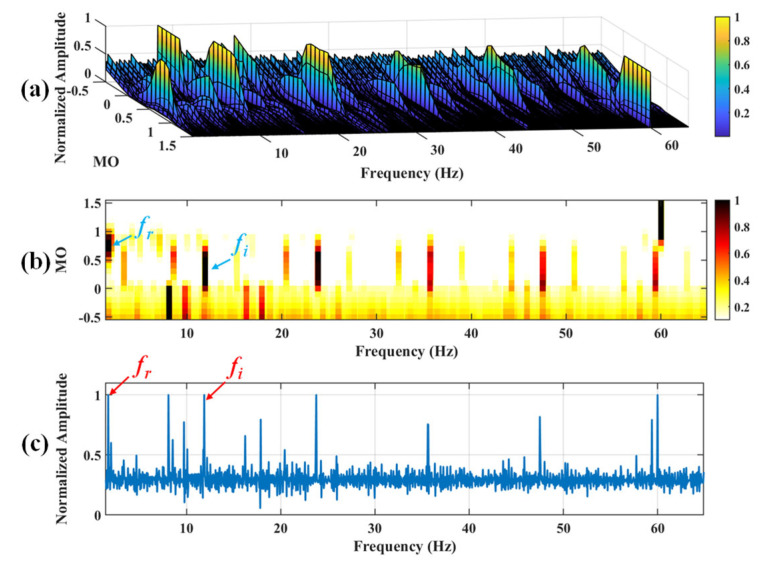
The demonstration of EMD-SAM. (**a**) The 3D plot. (**b**) The 2D plot. (**c**) The MSES.

Firstly, wavelet decomposition is applied to the raw signal to obtain the approximate part and the detailed part of the wavelet coefficients at levels 1 to 8, as shown in Figure 36a. The approximate part and the processed detailed part of the wavelet coefficients at levels 1 to 8 are obtained through threshold processing, as shown in Figure 36b. Then, the denoised signal is obtained through wavelet reconstruction. In this experiment, the raw signal is processed using the decomposition level (*J* = 8), the wavelet basis function (sym2), and the threshold selection (rigrsure). The soft thresholding rule is applied at each level. Figure 37 depicts the comparison between the raw signal and the denoised signal, showing that by utilizing wavelet threshold denoising, the noise interference of the raw signal is further suppressed. In the following, the proposed method is applied to the denoised signal, as shown in Figure 38. According to Figure 38b, the inner ring fault feature frequency, *f_i_*, can be clearly detected when −0.5 ≤ MO ≤ 0.7. According to Figure 38c, the fault feature frequency, *f_i_*, and its fourth harmonic can also be clearly observed in the MSES, which is sufficient to identify that it is an inner fault. For ease of comparison, Figure 39 shows the results of processing the raw signal utilizing SAM. As shown in Figure 39b, the fault feature frequency, *f_i_*, can be detected only when 0.1 ≤ MO ≤ 0.7. As shown in Figure 39c, the fault feature frequency, *f_i_*, cannot be effectively identified. The results obtained by replacing wavelet threshold denoising with VMD are shown in Figure 40. The results obtained by replacing wavelet threshold denoising with EMD are shown in Figure 41. Based on the above analysis, the proposed method can effectively identify the fault feature frequency, *f_i_*, when the rotating speed is 100 r/min.

In this paper, the Feature Factor of Envelope Spectrum (EFF) is utilized to characterize the fault feature intensity in the MSES. The larger the EFF index, the greater the proportion of the fault feature frequency component in the MSES. The results are shown in Table 5. Additionally, when using an i9-13900HX CPU, the computation times of different methods are presented in Table 6.

## 6. Conclusions

To solve the issue of difficult extraction of bearing fault features in low-speed, a low-speed bearing fault diagnosis method based on wavelet threshold denoising and SAM is proposed. Firstly, the raw signal is denoised by wavelet threshold denoising to reduce the interference of strong background noise, thereby obtaining the denoised signal. Secondly, the denoised signal is subjected to SAM to enhance the bearing fault impulses, thereby extracting the fault feature frequency. Finally, the proposed method is applied to both simulated signals and experimental signals. The experimental results demonstrate that the fault features of outer and inner rings can be effectively identified in low-speed. Although the proposed method can effectively extract fault features of the outer and inner rings in low-speed, some limitations still exist. The method does not achieve ideal results in low-speed fault diagnosis for ball and separator defects. In future research, we will conduct in-depth investigations into SAM-based low-speed fault diagnosis to extract fault features of ball defects, separator defects, and compound faults, while addressing the issue of robustness.

## Figures and Tables

**Table 1:** Parameters of bearings.

Parameter	Value
Defect on outer	0.3 × 0.05 (width × depth)
Defect on inner	0.3 × 0.05 (width × depth)

**Table 2:** The fault feature frequency of the bearing.

Speed	*f*_0_	*f_i_*
500 RPM	36.5956 Hz	55.0711 Hz

**Table 3:** Parameters of bearings.

Parameter	Value
Defect on outer	0.6 × 0.3 (width × depth)
Defect on inner	0.6 × 0.3 (width × depth)

**Table 4:** The fault feature frequency of the bearing.

**Speed**	***f*_0_**	***f_i_***
100 RPM	8 Hz	12 Hz

**Table 5:** The comparison of EFF index.

Speed	Fault Type	WTD-SAM	SAM	VMD-SAM	EMD-SAM
500 RPM	Outer fault	62.09	6.70	7.72	6.97
	Inner fault	53.27	9.41	8.70	32.60
100 RPM	Outer fault	36.28	18.31	20.67	14.23
	Inner fault	35.73	20.23	20.29	18.06

**Table 6:** Comparison of time calculation by different methods.

Speed	Fault Type	WTD-SAM	SAM	VMD-SAM	EMD-SAM
500 RPM	Outer fault	3.52 s	3.02 s	115.84 s	4.31 s
	Inner fault	3.89 s	3.16 s	34.33 s	4.53 s
100 RPM	Outer fault	3.94 s	3.28 s	49.30 s	4.89 s
	Inner fault	3.51 s	2.98 s	47.95 s	4.33 s

## Data Availability

The raw/processed data cannot be shared at this time. Due to the nature of this research, participants of this study did not agree for their data to be shared publicly.
